# The Proposed Bill to Regulate the Practice of Dentistry in the State of Maryland

**Published:** 1883-12

**Authors:** 


THE PROPOSED BILL TO REGULATE THE PRACTICE OF DENTISTY IN THE STATE OF MARYLAND.—The following Bill was adopted at the Meetings of the Maryland State Dental Association, held October 18th, and November 8th, 1883, and will be presented for the action of the State Legislature, which assembles in January 1884.

A BILL.

*Entitled an Act to regulate the Practice of Dentistry, and to protect the people against Empiricism in relatiou thereto, in the State of Maryland.*

---

Section 1. *Be it enacted by the General Assembly of Maryland*, That it shall not be lawful for any person or persons to engage in the practice of dentistry in the State of Maryland, unless said person or persons have graduated and received a diploma or diplomas from the faculty of a Dental College, or a Dental Department connected with a Medical University or Medical College, chartered under the authority of one of the

United States of North America, or from a foreign Government, or shall have obtained a license from a Board of Examiners, duly authorized and empowered by this Act to issue such license.

Sec. 2. *And be it enacted*, That the said Board of Examiners shall consist of five dental graduates, who are members in good standing of the Maryland StateDental Association, provided that said graduates have been practicing in the State of Maryland, for a term not less than three years; the said Board of Examiners shall be appointed by the Maryland State Dental Association at their next annual meeting, or so soon as they may deem it necessary, who shall serve for a term of three years, when their successors shall be appointed, and every third year thereafter, unless otherwise provided by law; the said Board shall have power to fill all vacancies in said Board, for unexpired terms.

Sec. 3. *And be it enacted*, That it shall be the duty of said Board: *first*, to meet annually at the time of meeting of the Maryland State Dental Association, or oftener, at the call of any three members of said Board; thirty days notice must be given at the annual meeting; *secondly*, to grant a license to any applicant who shall furnish satisfactory evidence of having graduated and received a diploma from any reputable and incorporated Dental College, or any Dental Department connected with a reputable and incorporated Medical College or University, without fee, charge, or examination; *thirdly*, to grant license to all other applicants who undergo a satisfactory examination before said Board; for which a fee of $5.00 shall be charged, the same fee to be applied by the Maryland State Dental Association for the expense of the Examining Board; *fourthly*, to keep a book, in which shall be registered the names of all persons licensed to practice dentistry in the State of Maryland.

Sec. 4. *And be it enacted*, That the book so kept, shall be a book of record, and a transcript from it, certified to by the officer of said Board having it in keeping, with the seal of said Board attached thereto, shall be evidence in any Court in the State.

Sec. 5. *And be it enacted*, That three members of said Board shall constitute a quorum for the transaction of business, and should a quorum not be present on the day appointed for their meeting, those present may adjourn from day to day, until a quorum is present.

Sec. 6. *And be it enacted*, That one member of said Board may grant a license to any applicant to practice dentistry, until the next regular meeting of the Board, when he shall report the fact, at which time, the temporary license shall expire; but such temporary license, shall not be granted by a member of the Board, after the Board has rejected the applicant.

Sec. 7. *And be it enacted*, That any person or persons who shall in violation of this Act, practice dentistry in the State of Maryland for a fee or a reward, shall be deemed guilty of a misdemeanor, and upon conviction thereof, shall be punished by a fine of $100.00 or not more than $500.00, and be imprisoned in jail not less than one month or more than one year; *provided*, that none of the provisions of this Act shall apply to regular licensed physicians, or those holding diplomas, and surgeons in practice, or dentists who have been in active practice for ten years prior to the passage of this Act.

Sec. 8. *And be it enacted*, That every person practicing dentistry in this State, shall within ninety days after the passage of this Act, register his name, together with his post office——, and the date of his diplomas or license, in the office of the Clerk of the Circuit of the county or Court of Common Pleas of the city in which he practices, and shall on the payment to such Clerk, a fee of fifty cents, be entitled to receive from him a certificate of such registration.

Sec. 9. *And be it enacted*, That all laws and parts of laws, in conflict with this Act be, and the same are hereby repealed.

Sec. 10, *Be it enacted*, That this Act shall take effect from the date of its passage.

